# Use of Fine-Needle Aspiration in the Evaluation of Breast Lumps

**DOI:** 10.4061/2011/689521

**Published:** 2011-06-21

**Authors:** Mulazim Hussain Bukhari, Madiha Arshad, Shahid Jamal, Shahida Niazi, Shahid Bashir, Irfan M. Bakhshi, Shaharyar

**Affiliations:** ^1^Department of Pathology, King Edward Medical University, Lahore 54000, Pakistan; ^2^Department of Pathology, Armed Forces Institute of Pathology, Rawalpindi, Pakistan; ^3^Salford Royal Hospital Trust NHS, University of Manchester, Manchester M13 9PT, UK

## Abstract

*Background*. A study was designed to see the role of fine needle aspiration cytology (FNAC) in palpable breast lumps. *Materials and Methods*. Four hundred and twenty five (425) patients came to the Department of Pathology King Edward Medical University, Lahore in four years for FNAC of their palpable breast masses from June 2006 to June 2010. FNAC diagnosis was compared with histological diagnosis to see the accuracy of fine needle aspiration cytology for neoplastic lesions. *Results*. There were 271/425 benign, 120/425 malignant, and 32/425 suspicious smears. Inadequate samples were repeated twice or thrice, and the degree of success was improved with consecutive repeating approaches. The frequency of inadequacy declined from 86 to 18, and 2 for first, second and third attempts, respectively. The number of repeats increased the diagnostic accuracy of aspirates which is statistically significant (*P* = .000). Invasive ductal carcinoma was the most commonly reported lesion with maximum incidence in the 4th, 5th, and 6th decades followed by invasive lobular carcinoma and other malignant lesions. The sensitivity, specificity, accuracy, negative predictive value, and the positive predictive value of FNAC was 98%, 100%, 98%, 100%, and 97%, respectively. *Conclusion*. FNAC serves as a rapid, economical, and reliable tool for the diagnosis of palpable breast lesions because the cytopathological examination of these lesions before operation or treatment serves as an important diagnostic modality.

## 1. Introduction

All breast lesions are not malignant, and all the benign lesions do not progress to cancer; however the accuracy of diagnosis can be increased by a combination of preoperative tests (like physical examination, mammography, fine-needle aspiration cytology, and core needle biopsy). These modalities are more accurate, reliable, and acceptable when compared with a single adopted diagnostic procedure despite of having their own technical limitations [1, 2].

“As fine-needle aspiration (FNA) has become a critical component in the investigation of palpable breast masses; false-negative diagnoses have become a major concern, prompting re-evaluation of the definition of specimen adequacy. Although cytopathologists agree that a number of parameters relate to the adequacy of an FNA specimen, there is no concsensus on the role of epithelial cell quantitation in the determination of an adequate FNA. To better understand the significance of epithelial cellularity, false-negative FNA samples from palpable breast lesions were reviewed” [3]. 

“Fine-needle aspiration (FNA) biopsy is an established and highly accurate method for diagnosing breast lesions.” The use of core biopsy (CB) is being increasingly advertised but its procedure is more cumbersome, expensive and time consuming as compared to FNA procedure [4–6]. Core Biopsy or tru cut needle biopsy is not widely used because of its complications, interpretation, and time-consuming results; therefore palpable breast lesions can be accurately diagnosed by triple test only (FNAC, physical examination and Mammography) [7]. 

“Although fine-needle aspiration (FNA) biopsy of the breast has been shown to be a safe and accurate technique, many surgeons question whether it is reliable enough to replace excisional biopsy. If FNA biopsy is followed by an excisional biopsy for confirmation, it would seem that the cost of diagnostic workup would be increased, but it has been seen that FNA biopsy is cost effective even when followed by an excisional or frozen section biopsy for confirmation. It is considered safe and reasonable to expand its use to smaller hospitals where the personnel may be initially less experienced with the technique” [8, 9].

It is considered a successful and less complicated procedure with excellent results; however the main factors influencing success should be considered before its procedure to increase its accuracy and these are “the aspirator, the small size of many cancers, and the occult nature of the lesions seen only on mammography” [10].

Fine-needle aspiration cytology (FNAC) is widely used in Pakistan as a reliable, rapid, cost-effective, complication free, and an accurate diagnostic modality for the evaluation or management of breast lumps. A study was conducted to see the usefulness of fine-needle aspiration (FNA) in screening of palpable breast masses at the Department of Pathology, King Edward medical University, Lahore, Pakistan.

## 2. Material and Methods

King Edward Medical University is the largest Medical Institute of Asia and Pakistan, comprising of four tertiary care hospitals with record numbers of yearly registered patients coming from all parts of the country. We have two programs at our institution: one is screening of all palpable lumps for early detection of malignancy and onsite diagnostic program for all referral cases waiting for surgery and treatment. 

A total of 425 patients came to the Department of Pathology in four years for FNAC of their palpable breast masses from June 2006 to June 2010. 

Out of these 425 patients, 338 biopsies were collected for comparison as inflammatory, and inadequate lesions were excluded. 

### 2.1. Patients Protocol

A designed proforma was used to collect the consent and data of patients. History of lactation and pregnancy was included in the proforma. All the lesions were followed for biopsy except inflammatory and inadequate samples. The inadequate lesions were advised a repeat FNAC. Both benign and malignant tumors were followed up. Patients were divided into groups, and their mean age was calculated.

### 2.2. Procedure for Fine-Needle Aspiration Cytology (FNAC)

A written consent was taken before performing the FNAC. FNA was done using a 23 gauge needle and 10 mL disposable syringe of Becton and Dickinson Pakistan (Pvt) for each prick and for each patient. No local anesthetic was used, and the needle was inserted into the palpable lesions, either once or twice depending upon the size of the nodule. FNA gave us cytological diagnoses, and we visualized at individual cells but cellular structures and not at macrotissue architecture like a histological specimen would. Cellular material was aspirated into a syringe and expelled onto slides. Four to five slides were prepared for each patient. A small or medium-sized drop of aspirate was put near the frosted end of a slide that was placed on a table. A second slide was used to spread the aspirated material in the same manner used to prepare a peripheral blood smear. All the smears were wet fixed in 95% methanol, and the air dried smears were stained with two stains Hematoxylin & Eosin (H&E), and Papanicolaou stains. The procedure was done within one hour, and the reports were signed out within 2-3 hours.

#### 2.2.1. Four Groups Were Defined for the FNAC Diagnosis

  (1) Inadequate
  (2) Non neoplastic lesions
  (3) Suspicious of malignancy
  (4) Positive for malignancy.

NB: in the calculations, groups 3-4 were malignant.

### 2.3. Preparation of Cell Blocks

Remaining aspirates were used for cell blocks, by putting 10% formalin in the syringes used for FNA.

### 2.4. Procedure for Histopathology

Inflammatory (85) and Inadequate (2) smears were not followed for their biopsies. Remaining 338 (benign smears, suspicious, and malignant) smears were followed for biopsies (76 mastectomies and 262 lumpectomies). These biopsy specimens were fixed in 10% formalin for 24 hours and then grossed in the Department of Pathology by consultant histopathologists. The gross and cut section findings were noted. Several bits were taken from appropriate sites for processing and paraffin embedding. From each block, sections were cut at 4-5 microns thickness and stained by H&E.

### 2.5. Criteria for Selection of Patients

Inclusion criteria.

1. All females with unknown primary diagnosis of breast mass.
2. Patients consented for inclusion in study according to designed proforma.

Exclusion criteria.

1. Patients with recurrent malignancy.
2. Patients who underwent FNAC but did not undergo subsequent histopathological diagnosis.
3. Patients in whom FNAC was either acellular or nondiagnostic or inflammatory.
4. Past or current chemo-therapeutic or prevention treatment.
5. Male patients with breast cancer and gynecomastia.

### 2.6. Statistical Analysis

Data was computerized with window SPSS version16. Specificity, sensitivity, accuracy, and predictive values were calculated, *P* values were also calculated, while correlation was seen by Pearson's correlation curve.

## 3. Results

A total 425 fine-needle aspirates (FNAs) were carried out over a period of four years in the Department of Pathology. There were 85 inflammatory lesions and 2 smears inconclusive. Remaining 338 (benign smears, suspicious, and malignant) smears were followed for biopsies (76 mastectomies and 262 lumpectomies). There were total of 256/425 (60%) benign lesions comprising of 85 (20%) inflammatory aspirates and 171(40%) benign proliferative lesions. Cytological diagnoses of other aspirations were reviewed, and lesions were classified into four diagnostic classes revealing 171 benign proliferative lesions, 131 malignant, 36 suspicious, and 2 inadequate smears (Table 4). 

### 3.1. Followup of Inadequate Smears

**Figure 9:**
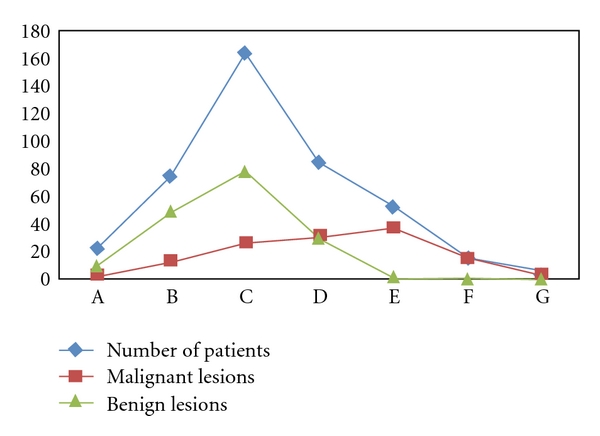
Correlation between Benign and malignant lesions in different Age groups of patients presented with lump breast. Note: A positive correlation was observed between Benign and malignant lesions (*r* = 0.95, *P* < .0001).

Inadequate samples were repeated twice and degree of success was improved with two consecutive approaches with an interval of 24 hours in subsequent repeats. There were 86, 18, and 2 inadequate smears in first, second, and third approach. The number of repeats increased the diagnostic accuracy which is statistically significant (*P* = .000). There were maximum lesions during the reproductive age groups, and most of these were benign. Maximum malignant lesions were seen in older age groups. This relationship was statistically significant when compared by Pearson correlation curve. A positive correlation was observed between benign and malignant lesions (*r* = 0.95, *P* < .0001) (Table 1, Figure 9). The age of the patients ranged between 16 to >70 years. The youngest patient diagnosed as invasive ductal carcinoma (IDC) was seen at 16 years of age. In our study, most of the benign lesions were reported (29.2% & 46%) in 3rd and 4th decades of life, while maximum numbers of malignant lesions (23.6 and 29%) were reported in 5th and 6th decades (Table 1).

There were 23.6%, 30.6%, 17.6%, 14%, 9%, 2.3%, and 2.3% lesions of acute suppurative mastitis, acute mastitis, non-tuberculous mastitis, chronic nonspecific mastitis, duct ectasia, tuberculous mastitis, and fat necrosis, respectively. The diagnosis of these lesions was made by cytology and histology of cell blocks (Table 2).

In benign proliferative breast lesions, we were very conscious about over-diagnosis, therefore our reporting style was as “smear negative for malignant lesions”, and for further analysis we advised an excision biopsy. For definite diagnosis our reports were limited to Fibroadenoma, fibrocystic change disease, benign proliferative disease, and Phyllodes tumors. For uncertain categories we only reported “benign proliferative lesions”. This class of diseases constituted the largest group of lesions with maximum incidence in the 3rd, 4th and 5th decades of life (Tables 1 and 3). 

Invasive ductal carcinoma was the most common malignant lesion reported in our study with a maximum incidence in the 4th, 5th, and 6th decades, followed by invasive lobular carcinoma and other malignant lesions (Table 5). Following histopathologic correlation with FNA, we calculated the sensitivity, specificity, and positive predictive values. 

There were 4 false positive cases and no false negative cases in this study. False positives were observed in the suspicious lesions. 

False positives noted mainly in the interpretation of suspicious smears or with atypical features, were due to uniformly enlarged nuclei with prominent nucleoli, occasional marked nuclear enlargement, and moderate pleomorphism seen in fibrocystic disease or fibroadenoma. Regarding benign proliferative and malignant lesions no false positivity was seen. Therefore, in the this study, the sensitivity was 100%, specificity 98%, accuracy 98%, negative predictive value 100%, and the positive predictive value was 97% (Table 5). 

All the FNAC smears are compared with histopathology and the details given in Figures 1, 2, 3, 4, 5, 6, 7, and 8.

## 4. Discussion

Fine-needle aspiration cytology is widely used in the diagnosis of breast cancer because it is an excellent, safe, and cost-effective diagnostic procedure. One can get on site immediate report with minimal cost using inexpensive equipments and a simple technique. The most significant advantage of FNAC is the high degree of accuracy, rapid results, and a less invasive procedure than a tissue biopsy. FNAC of the breast can reduce the number of open breast biopsies [11–14].

The frequency of inadequate cases are variable is different studies ranging from 0 to 57.2% depending on various factors. The main causes for inadequate smears may be due to either lack of technical experience in performing FNA, preparation, and fixation of smears. FNA of ill-defined masses like or lesions with hyalinization and deeply located lumps may also be contributed to the inconclusive diagnosis [15, 16]. 

Many inflammatory breast lesions create confusion as these are presented as a palpable mass. “Mammographic, sonographic, and magnetic resonance imaging findings may not always distinguish some of the benign lesions like duct ectasia, fat necrosis from a malignant lesion.” Fine-needle aspiration (FNA) is a well-accepted diagnostic modality and procedure for the diagnosis of inflammatory swellings of breasts. We are using this technique in such lesions, and results are variably accepted by our consultants and clinicians with varying degrees of acceptance rates, accuracy, and results. Fine-needle aspiration is the most accurate diagnostic modality for these lesions and cell blocks accentuate the reliability of the diagnosis in these benign inflammatory and curable lesions without requirement of excision biopsy or other second-line investigations. In this study, these were reported as benign inflammatory diseases and their histopathologies was followed and were further categorized into different lesions. Cell blocks were prepared after making the required smears and were processed for histopathology [17–19]. 

There were 85 (20%) cases of benign inflammatory lesions, and the majority of these were of acute and chronic mastitis. “Granulomatous mastitis is a rare chronic inflammatory breast lesion that mimics carcinoma clinically and radiologically” [19–21]. There were 2 (2.3%) cases of tuberculosis; definitive diagnosis of the tuberculous mastitis was based on identification of typical histological features under microscopy and detection of tubercle bacilli on Ziehl-Neelsen stain. There were 8 (9%) patients of duct ectasia whose histological diagnosis was based on observing dilatation of major ducts which contained eosinophilic granular secretions and foamy histiocytes both within the duct epithelium and in the lumen. Two cases of fat necrosis were also reported on histopathology, characterized by “anucleated fat cells surrounded by histiocytic giant cells and foamy macrophages”. Our findings are consistent with Nemenqani et al. that primary tuberculous mastitis is very rare in Pakistan [19]. 

FNAC has some pitfalls in the diagnosis of Fibrocystic disease (FCD), adenosis, epithelial hyperplasia with or without atypia, apocrine metaplasia, radial scar, and papilloma “[22]. Fibroadenoma and these benign lesions are more common in our setup. Various types of adenosis have also been described, of which sclerosing adenosis and microglandular adenosis merit detailed description and most of these lesions mimic malignant lesions” [23].

In this study, 338 FNA aspirations were correlated with histopathology to evaluate the diagnostic sensitivity, specificity, and accuracy of this diagnostic modality. Among these lesions 171 (40%) were benign proliferative lesions. In our center, we further categorized these lesions into three main groups; namely fibroadenoma, fibrocystic disease, and benign proliferative diseases. The spindle cell lesions were diagnosed as benign Phyllodes on cytology reports. These results were confirmed by histopathology from the cell blocks and tru-cut biopsies. In our study, no malignancy was seen while few discrepancies were seen in making final categories like out of 70 FNAC diagnosed FA, there were 60, 6,3, and 1 FA, FCD (When there were mixture of cysts, fibrosis, and proliferating ductal epithelium), FAN (overgrowth of both fibrous stroma, and of epithelial elements, i.e., ducts and lobules, in differing proportions), and benign Phyllodes on histopathology. From 90 FCD, there were 70 FCD on histopathology while other were 10, 5, 3, 1, and 1 cases of FA, FAN, florid epithelial hyperplasia (FEH), atypical epithelial hyperplasia (AEH), and benign Phyllodes, respectively.

In our experience, FNAC results are more reliable regarding malignant lesions; however the category of “Suspicious for Malignant Lesions” needs histopathological evaluation before performing surgical measures. Self-assessment, mammography, and tru-cut biopsy may help in the accuracy of these lesions [2]. 

It is widely accepted that FNA is a less traumatic and easy technique than core needle biopsy because we repeated the FNAC in case of inadequate smears without any delay, difficulty, trauma, and getting highly accurate results. This statement is not applicable for open biopsy as it is a time consuming and cumbersome technique which requires fixation, processings, staining and so forth. It is also expensive procedure costing Rs 700 (9.5 USD) as compared to Rs 200 (2.5 USD) for each FNA while it is also expensive in advanced countries. In a study Rubin et al. has mentioned a saving of 1000$ with this cost effective procedures [4]. In our study the accuracy of FNA aspiration was increased by repeating the process within 24 hours and was found to be significant (*P* = .000). There were many reasons for inadequate smears like size, type of lesions, experience of the technical staff, and cooperation of patients in our study. 

We have a proper and permanent FNAC modality room, onsite service, technical trained staff, expert consultants, and a large number of postgraduate students available for FNA service. We provide 90% free services; however, our charges are 1.2 USD for the affording cases. We have direct interaction with the clinicians, patients, and our laboratory technical staff. In our study, no false negative cases were reported when compared with histopathology.

Only four cases were observed as being false positives (1.7%). False positives were interpreted as “suspicious for malignancy” that were later on reported as benign proliferative lesions on histopathology. All benign and malignant reported aspirates showed 100% accuracy. In this study the sensitivity was 100%, and the specificity was only 98% because of false positives when compared with the histological reports. 

There is a wide range of false results regarding the credibility of FNAC. The rate of false negatives, false positives varied from 0% to 10% in various studies [4, 24, 25]. Our results are consistent with our previous study and other studies in Pakistan and other areas. “The overall results in our previous study for sensitivity, specificity, accuracy, PPV, and NPV were 97%, 100%, 97%, 100%, and 87%, respectively” [2]. The reasons for such a large range of variable results are multifactorial and the main factors being the small number of cases of FNA published, lack of onsite service, and coordination between surgeons, radiologists and pathologists [24, 26].

## 5. Conclusion

The cytological examination of breast lesions prior to surgical treatment serves as a rapid, economical, and valuable diagnostic tool. Adhering to the principle of “Triple test,” and acquisition of technical, observational, and interpretative skills will further enhance the diagnostic accuracy of proliferative conditions with atypia or suspicious lesions of breast. 

## 6. Recommendations

1. Proper training and skilled courses should be arranged for the technical staff because it increases the reliability and accuracy of the test.
2. No overemphasis should be made in the reporting of FNA, and very careful strict criteria should be adapted like advised repeat FNAC, cell block preparation, biopsy or correlation with clinical findings and so forth. Three tier system should be used instead of 5 classes of the breast cytology, for example, benign, suspicious, and malignant. FNAC should be repeated when inadequate and improper smears are prepared.
3. When the results are obviously benign, patients should be reassured and can be prevented from undergoing unnecessary surgery while in the case of a clearly malignant smears, surgery and other treatment should be started without any delay.
4. For the gray areas, for example, suspicious and inadequate smears, either repetition of the FNA or a surgical biopsy should be recommended.

##  Conflict of Interests

There is no conflict of interests. 

## Figures and Tables

**Figure 1:**
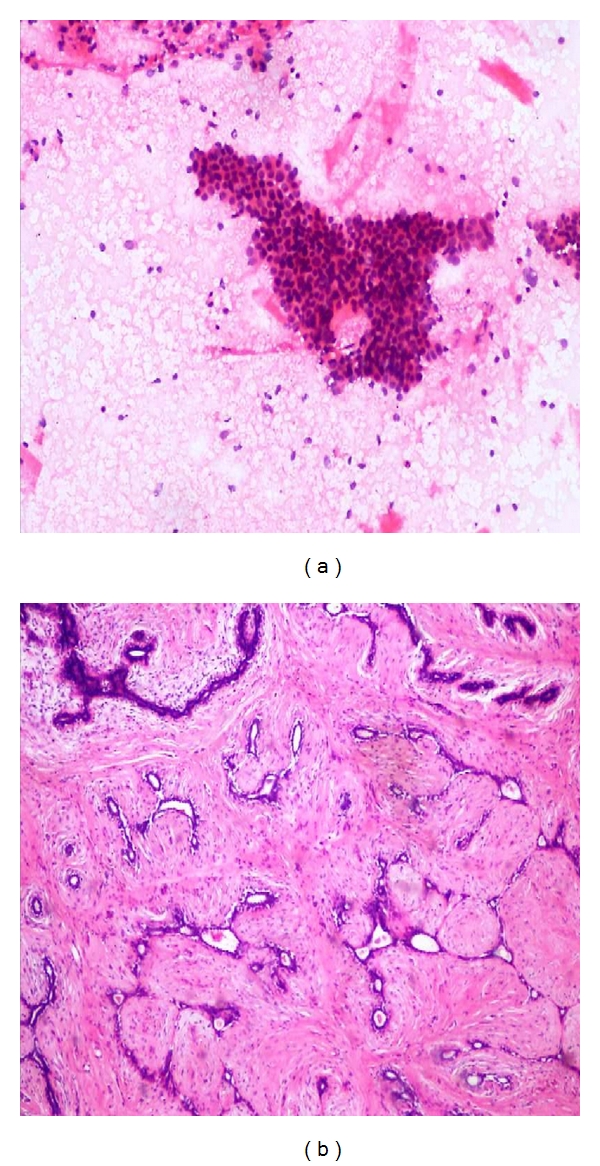
(a) Photomicrograph on FNAC of a benign smear (H&E stain 10x) of Fibroadenoma showing sheet of regularly arrange epithelial cells (Stag horn pattern) and numerous bare nuclei. (b) Comparison on Histopathology (10x H&E) of fibroadenoma breast.

**Figure 2:**
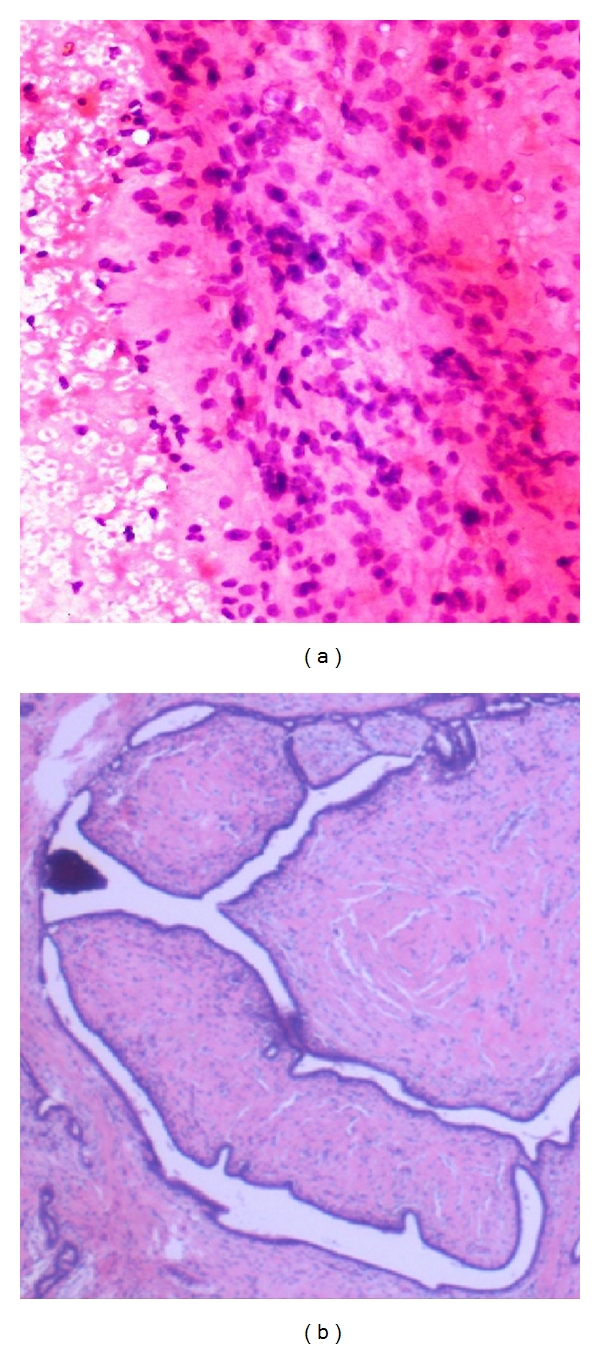
(a) Photomicrograph on FNAC of a benign smear of Benign Phyllodes (H&E stain 20x) showing cellular smear of spindled stromal cells. (b) Comparison on Histopathology of Benign Phyllodes.

**Figure 3:**
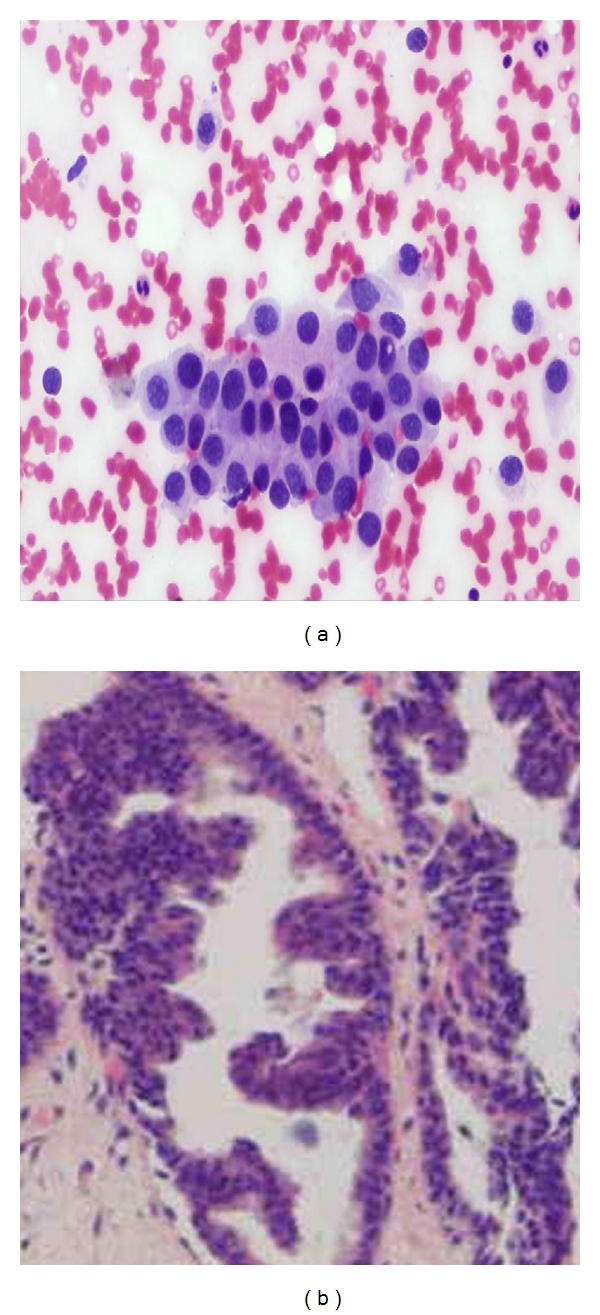
(a) Photomicrograph on FNAC of a susp**i**cious smear (H&E stain 40x) of breast showing increased cellularity with mild pleomorphism and discohesion but lacking other features of malignancy. (b) Comparison on histopathology of atypical ductal hyperplasia.

**Figure 4:**
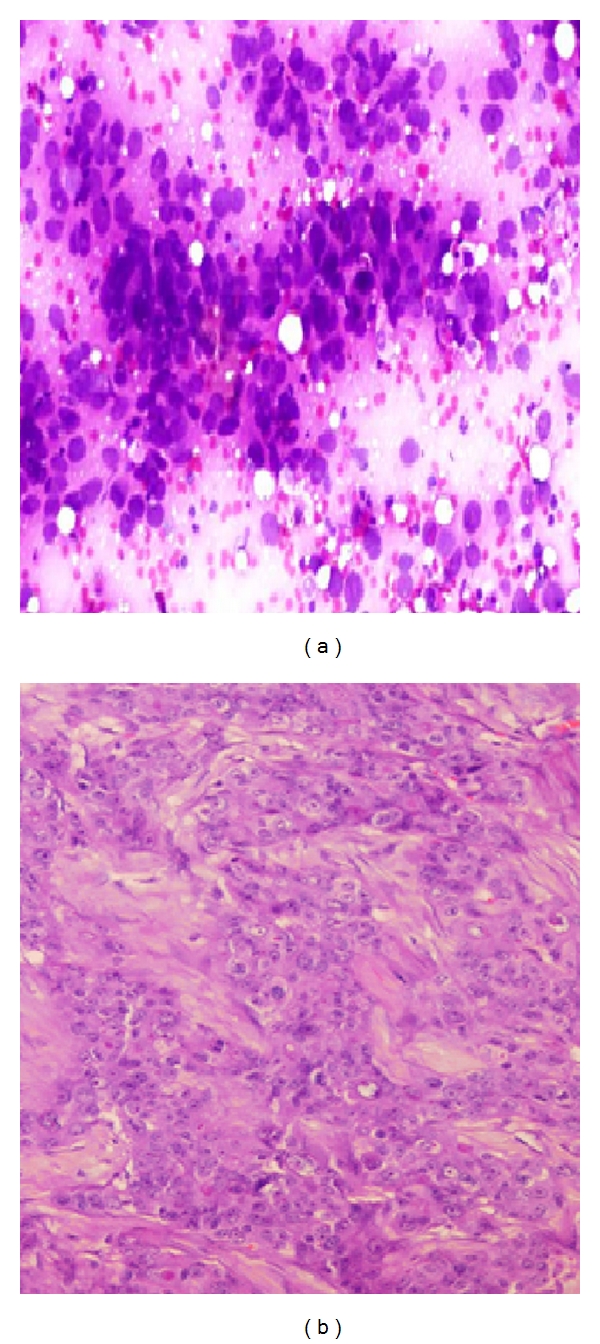
(a) Photomicrograph on FNAC of a malignant smear of ductal carcinoma (40x Pap stain) showing variation in nuclear shape and size, with decreased intercellular cohesion and dirty background (b) Comparison on histopathology of invasive ductal carcinoma

**Figure 5:**
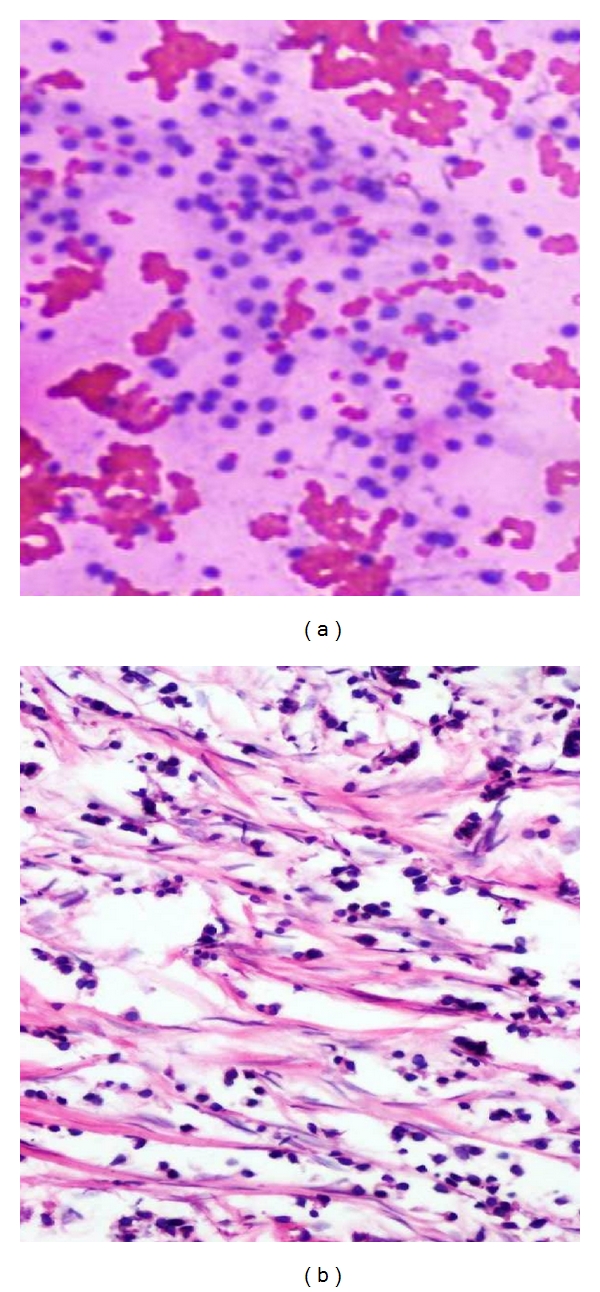
(a) Photomicrograph on FNAC of a malignant smear's depicting cytological features of lobular carcinoma (20x H&E stain). Groups of small, round, uniform cells with distinct cell membrane and discohesion. (b) Comparison on histopathology showing uniform cells arranged in alveolar pattern of lobular carcinoma.

**Figure 6:**
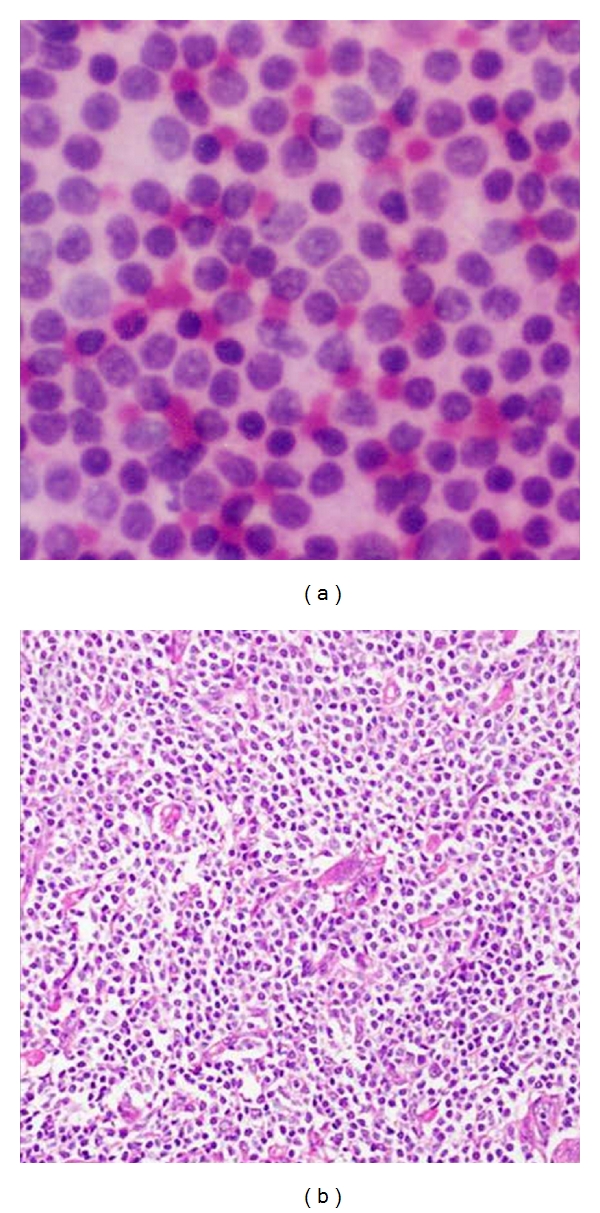
(a) Photomicrograph on FNAC of a malignant smear of Non-Hodgkin Lymphoma (H&E stain 40x). Sheets of noncohesive cells with scanty cytoplasm. (b) Comparison on histopathology of non Hodgkin's lymphoma (40x H&E).

**Figure 7:**
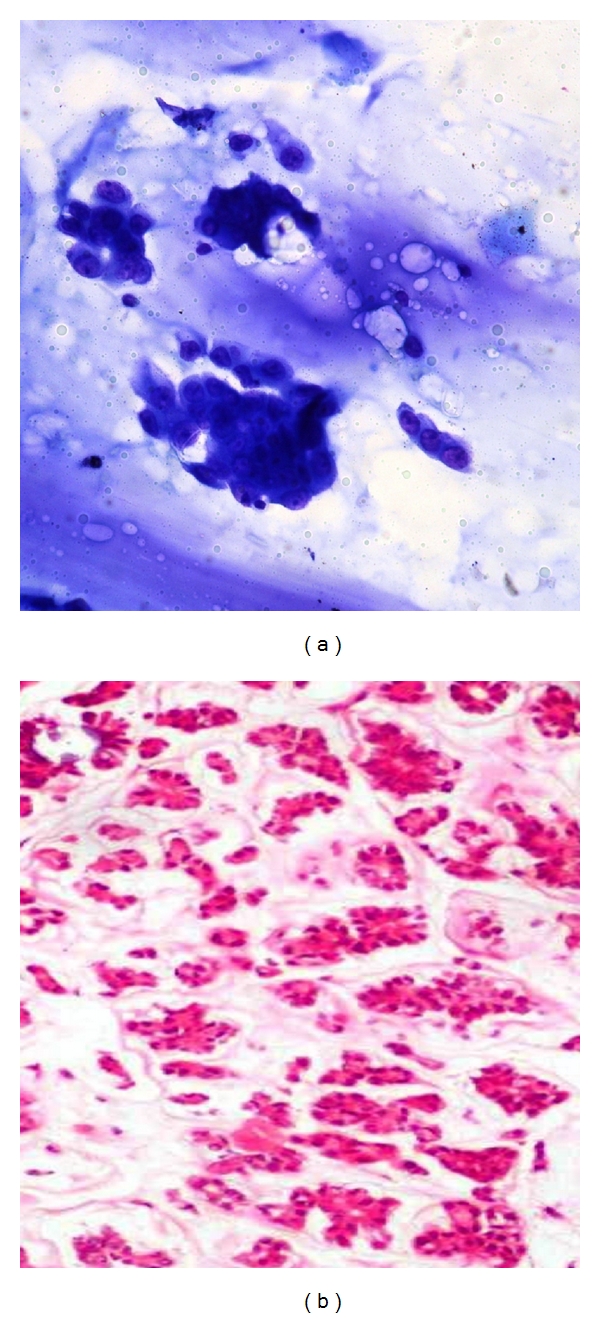
(a) Photomicrograph on FNAC of a malignant smear's of colloid carcinoma (20x Giemsa stain) with loose clusters of epithelial cells against abundant mucin. (b) Comparison on histopathology of mucinous carcinoma breast (10x H&E).

**Figure 8:**
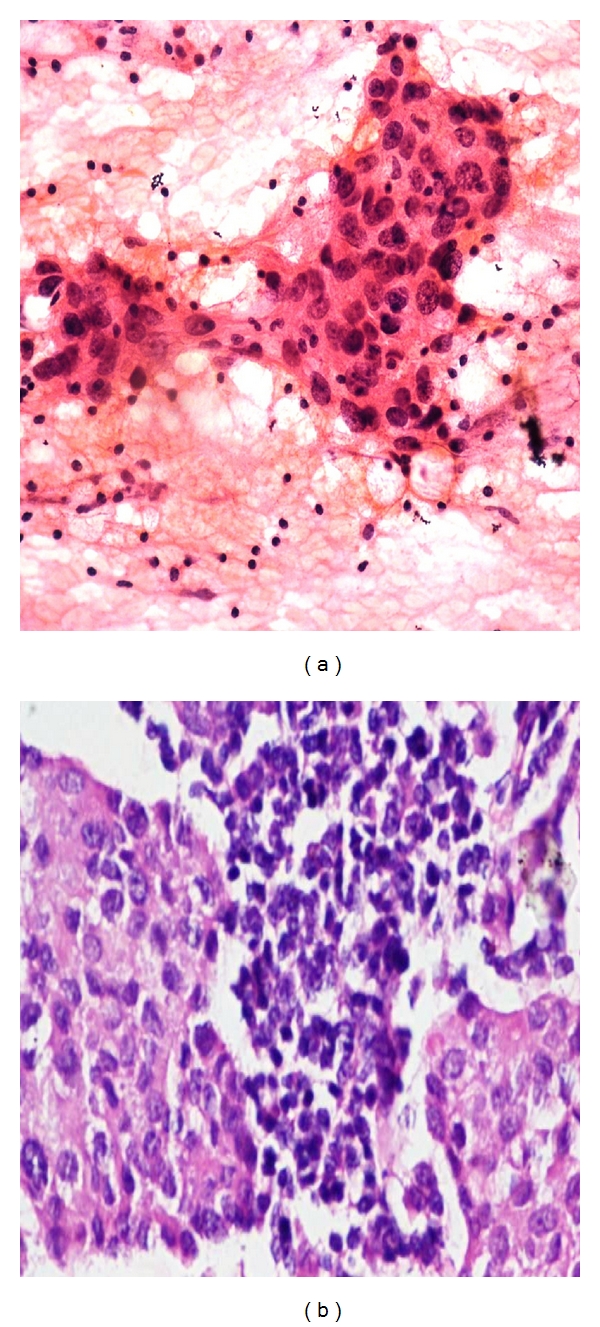
(a) Photomicrograph on FNAC of a malignant smear of medullary carcinoma (H&E stain at 40x) showing large to medium sized cells with large nucleoli with syncytial pattern against lymphoplasmacytic cells. (b) Comparison on Histopathology of Medullar carcinoma Breast (40x H&E).

**Table 1:** Age of the patients presenting with lump breast.

Age in years (*n* = 425)	Inadequate	Inadequate on first repeat	Inadequate second repeat	Benign lesions		Suspicious for malignancy	Malignant
				Inflammatory	Benign proliferative lesions		
A 16–20 (*n* = 23)	4	1	0	4 (4.7%)	10 (5.8%)	6 (16.6%)	3 (2.3%)
B 21–30 (*n* = 76)	12	2	0	10 (11.8%)	50 (29.2%)	3 (8.3%)	13 (10%)
C 31–40 (*n* = 166)	27	5	1	52 (61%)	79 (46%)	7 (20%)	27 (20.6%)
D 41–50 (86)	23	4	0	14 (16.5%)	30 (17.5%)	11 (30.5%)	31 (23.6%)
E 51–60 (*n* = 53)	17	5	1	5 (5.8%)	2 (1.2%)	7 (20%)	38 (29%)
F 61–70 (*n* = 16)	2	1	0	0	0	1 (2.8%)	15 (11.5%)
G <70 years (*n* = 5)	1	0	0	0	0	1 (2.8%)	4 (3%)

Total	86	18	2	85 (20%)	171 (40%)	36 (8.4%)	131 (31%)

Note: the number of repeat increased the diagnosis of aspirates which is statistically significant (*P* = .000).

**Table 2:** Comparison of distribution of inflammatory lesions.

Inflammatory Lesions (*n* = 85)	Cytology	Histopathology
Acute suppurative Mastitis	20 (23.5%)	Excluded
Acute mastitis	26 (30.5%)	Excluded
Non-tuberculosis granulomatous mastitis	15 (17.6%)	Excluded
Chronic nonspecific mastitis	12 (14%)	Excluded
Duct ectasia	8 (9%)	Excluded
Tuberculosis	2 (2.3%)	Excluded
Fat necrosis	2 (2.3%)	Excluded

**Table 3:** Comparison of distribution of inflammatory lesions.

Benign Lesions on cytology (*n* = 171)	Cytology	Histopathology						
		FA	FSD	FAN	FEH	AEH	BPh	Malignant
Fibroadenomas	70 (41%)	60	6	3	0	0	1	0
Fibrocystic disease	90 (52.6%)	10	70	5	3	1	1	0
Benign proliferative diseases	6 (9%)	0	0	2	3	0	1	0
Benign phyllodes	5 (3%)	2	0	0	0	0	3	0

Total	171	72	76	10	6	1	6	0

FA: fibroadenoma, FCD: fibrocystic disease, FAN: fibroadenosis, AEH: atypical epithelial hyperplasia, FEH: florid epithelial Hyperplasia, and BPh: Benign Phyllodes.

**Table 4:** Histopathological diagnosis of suspicious and malignancy smears.

Cytology	Histopathology											
	Malignant results									Benign results		
	DCIS	IDC	LCIS	ILC	MC	MDC	MTC	Lymph	MPhy	FA	FAN	SCA
Suspicious for malignancy (*n* = 36; 8.4%)	15	10	4	4	0	0	0	0	0	1	2	1
Malignant lesions (*n* = 131; 30.8%)	4	100	2	16	2	1	1	1	4	0	0	0

FA: fibroadenoma, FAN: fibroadenosis, SCA: sclerosing adenosis, DCIS: ductal carcinoma Insitu. IDC: invasive ductal carcinoma, LCIS: lobular carcinoma In situ, ILC: invasive lobular carcinoma, MC: mucinous carcinoma, MDC: medullar carcinoma, MTC: metaplastic carcinoma, Lymph: lymphoma: MPh: malignant phyllodes.

**Table 5:** The Diagnostic accuracy of FNAC in histologically correlated cases.

FNAC result	Histopathological diagnosis		Total
Positive for malignancy	163 (TP)	4 (FP)	167
Negative for malignancy	0 (FN)	171 (TN)	171

Total	163	175	338

Sensitivity: 100%, Specificity = 98%, Accuracy = 98%, PPV = 97%, NPV = 100%.
